# Feasibility of Augmented Reality-Based Cognitive Training for Older Adults: The MarketMind AR Approach

**DOI:** 10.3390/s25072081

**Published:** 2025-03-26

**Authors:** Konstantinos Kakoutopoulos, Emmanouil Drakakis, Anastasia Papadopoulou, Christos Goumopoulos

**Affiliations:** Information & Communication Systems Engineering Department, University of the Aegean, 83200 Samos, Greece; kakoutopoulos@gmail.com (K.K.); edrakakis@aegean.gr (E.D.); anastpapado@gmail.com (A.P.)

**Keywords:** augmented reality, serious games, cognitive training, elderly, system usability scale (SUS), game experience questionnaire (GEQ), unified theory of acceptance and use of technology (UTAUT)

## Abstract

**Highlights:**

**What are the main findings?**

- MarketMind AR showed good usability and high engagement, with participants reporting competence, immersion, and low frustration.
- Players improved performance over time, with faster completion, higher scores, and better PIN recall, suggesting cognitive benefits.

**What is the implication of the main finding?**

- AR-based cognitive training is cost-effective and accessible, using smartphones/tablets and built-in sensors instead of expensive VR headsets.
- The familiar supermarket setting enhances engagement and usability, supporting intuitive and long-term use.

**Abstract:**

The aging population increases the need for accessible interventions for cognitive training of the elderly to preserve cognitive health. Serious games have been widely used for this purpose, with many existing applications leveraging virtual reality (VR) technology. In contrast, this study explores the potential of augmented reality (AR) for cognitive training. The literature review shows that cognitive training interventions typically employ supermarket-themed serious games that are used extensively in such interventions. MarketMind AR is a supermarket-themed serious game that was created to train memory, attention, and executive function using mobile phone sensors such as cameras, accelerometers, and gyroscopes to interact and recognize the environment. Fifteen older adults participated in a three-attempt trial and completed the System Usability Scale (SUS), the in-game Game Experience Questionnaire (iGEQ), and an adapted version of the Unified Theory of Acceptance and Use of Technology (UTAUT) questionnaires. Qualitative interviews and in-game data (e.g., completion times, PIN recall) were also examined. The results indicated that participants had a positive experience, confirming ease of use, immersive appeal, and perceived cognitive benefits, despite some difficulties with environment scanning and object detection. The results provide evidence that an AR supermarket game leveraging mobile sensors has the potential to be an effective cognitive training tool for older adults.

## 1. Introduction

By 2050, the global population aged 65 and older will more than double to 1.6 billion, with those aged 80 and above growing even faster [1]. In particular, life expectancy in 1990 was 66.5 years for women and 61.5 years for men, while by 2050, it is expected to reach 79.8 and 77.2 years, respectively, while in Europe and North America, it is 86.1 and 83.8 years. This results in population aging, a process in which the proportion of older people in the general population increases and that of younger people decreases [2]. This demographic transition is expected to drive a sharp increase in cognitive disorders, with global dementia cases projected to triple to 152 million during the same period [3]. These trends highlight significant challenges, such as the rising healthcare and care-giving costs, disproportionately affecting women and disadvantaged groups [1,4].

Mild Cognitive Impairment (MCI) is a transitional stage between normal aging and more serious diseases such as Alzheimer’s in which there are noticeable declines in memory, language, and thinking skills beyond those expected due to aging. MCI is influenced by diverse factors, including biological, environmental, genetic, lifestyle, and comorbidities. Biological changes such as cerebral hypoperfusion, hypometabolism, brain atrophy, and the presence of amyloid plaques and tau tangles contribute to cognitive decline [5]. Environmental factors, including physical activity, a healthy diet, moderate alcohol consumption, and healthy weight, are associated with reduced risk, while education level, socioeconomic status, and urban versus rural living also play roles by impacting access to healthcare and social opportunities. Lifestyle habits like smoking and excessive alcohol consumption further affect MCI risk.

Serious games and augmented reality (AR) are increasingly utilized in cognitive training and cognitive rehabilitation to enhance engagement and effectiveness [6]. Serious games, designed for therapeutic purposes, leverage interactive elements to make training and rehabilitation enjoyable and impactful. Furthermore, AR games blend real-world and virtual elements, leading to deeper player engagement. These technologies are particularly beneficial for older adults, as they promote active participation and can simulate real-life scenarios for cognitive training.

This study evaluates the challenges of using AR serious games for cognitive training in the elderly. Additionally, it explores technology acceptance of an AR supermarket game designed for cognitive training. In particular, the purpose of this study is to present and evaluate MarketMind AR, a mobile AR supermarket game, as a cognitive training tool for older adults. It focuses on assessing the usability and the engagement of AR-based cognitive training and determining whether AR can serve as a practical and accessible alternative to other immersive technologies like virtual reality (VR), which require expensive headsets. Mobile AR utilizes cheaper devices like smartphones and tablets, making it a more accessible solution for older adults.

Daily activities based serious games and more specifically supermarket-themed games, like MarketMind AR, are well-suited for cognitive training because they simulate real-world tasks that engage multiple cognitive functions such as memory, attention, and executive function. This study evaluates how older adults perceive the usability and accessibility of MarketMind AR and offers an initial exploration of whether this type of game is an acceptable and practical approach for cognitive training.

The results of the evaluation study performed demonstrate that MarketMind AR is an effective cognitive training tool for elderly people, with good usability, good game experience, and high acceptance by users. By employing a mixed quantitative (SUS, iGEQ, UTAUT) and qualitative methodology, the study assessed usability, engagement, and acceptance. The usability test reported a median SUS score of 77.5, indicating the success of the intuitive design [7]. Participants reported high engagement, immersion, and competence, and low frustration, in the iGEQ questionnaire. Results from the UTAUT model suggest that ease of use and facilitating conditions were key factors in user acceptance, while examination of game session data indicated performance improvements in completion times and memory tasks, suggesting cognitive benefits. Qualitative comments corroborated the contribution of the everyday supermarket setting to engagement, highlighting the accessibility of MarketMind AR as a low-cost alternative to VR-based cognitive training.

## 2. Research Background

The use of serious games as a tool for promoting cognitive, physical, and social well-being among the elderly has gained significant attention in recent years, with studies showing their potential in improving cognitive abilities [8] and enhancing physical health through exergames [9]. Among the various game concepts, those simulating everyday activities, such as visiting a supermarket or engaging in market-like shopping tasks, are familiar and relevant to daily life. These games provide an engaging environment that can be used for both assessing and improving cognitive abilities, including decision-making, memory and motor skills in older adults. Immersive technologies, including VR and AR, enhance engagement by creating realistic and interactive experiences that improve both involvement and perceived authenticity.

Reference [10] evaluated the acceptance and usability of Virtual Supermarket, an immersive VR application developed for cognitive training in older adults with MCI or subjective cognitive decline. The serious game that was developed with the Unity engine and deployed on the HTC Vive Pro headset (which uses two infrared cameras as tracking sensors) requires users to perform a shopping task in a virtual supermarket. Participants select items from shelves based on a shopping list and place them in a cart using a motion-tracked controller. They also complete a payment task at a virtual cash register by selecting the correct amount of money. The study found the VR system to be well accepted, making it a promising tool for cognitive training.

In [11], the authors developed a VR-based serious game called the “Virtual Supermarket” that uses machine learning for screening early-stage neurocognitive impairments such as MCI and early Alzheimer’s disease. The serious game was created using the Unity engine and designed for use on HTC Vive headset with HTC Vive Lighthouse. In the game, the users are asked to memorize a shopping list, navigate through the virtual store to collect items, and complete a payment task at checkout. These tasks are designed to assess memory, planning, and executive functions by tracking users’ behavior in an immersive virtual environment. The study found that this method is effective, non-invasive, and cost-efficient for the early-stage assessment of cognitive impairments. 

Another immersive VR serious game for cognitive assessment and early detection of MCI, the “Immersive Virtual Reality Supermarket Cognitive Assessment Program” (IVRSCAP), is described in [12]. IVRSCAP was built using the Unity engine and was deployed on the Oculus Quest 2 HMD, utilizing its built-in motion sensors and the handheld controllers to allow users to interact with the virtual world. The main task simulates a supermarket environment where users must navigate, find items based on a shopping list on the shelves, and complete the transaction at a virtual checkout, supplemented by other mini-game tasks. The game is designed to assess multiple cognitive domains, including attention, memory, spatial navigation, executive functions, and processing speed. The proposed algorithms were found to be effective in differentiating MCI individuals and healthy elderly individuals with high accuracy and precision.

Reference [13] presented a supermarket-themed serious game for the cognitive training of elderly with MCI. The game was developed in Unity and designed to be deployed on Android tablets. The game consisted of four core exercises designed to stimulate key cognitive functions like memory and executive functions through tasks such as memorizing a shopping list, recalling objects, making payments, and organizing purchases. The usability study of the game showed that it was well received, entertaining, and with potential to be an effective tool for cognitive training.

A virtual supermarket exergame, developed in Unity, was implemented in both a WebGL-based non-immersive version (for PC browsers) [14] and an immersive VR version (using an HTC Vive headset with HTC Vive Lighthouse) [15]. The game is designed for the cognitive assessment of elderly individuals and simulates a virtual supermarket where users must complete tasks such as finding and selecting items from shelves based on a shopping list, managing the shopping basket, and completing the payment process by choosing the right banknotes and coins. The study for the immersive version suggested its potential for both cognitive assessment and training, while the WebGL version was primarily designed for cognitive assessment without requiring specialized hardware.

Reference [16] described the GAME2AWE platform, which features a range of virtual and augmented reality activities. One of the AR activities for Android devices involves purchasing bags of seeds displayed on a four-sided shelf. Players must walk around the shelves, locate the required bags of seeds by aiming their device’s camera at them, and then complete the purchase by selecting the correct payment amount. These AR activities were developed using Unreal Engine 4, designed to help users practice cognitive skills while engaging in physical activity.

Table 1 summarizes the reviewed studies, the game types, and equipment used for serious games. Most studies explored the use of VR, both immersive and non-immersive, for cognitive training and assessment in supermarket-themed serious games. However, only one study incorporated a market-related task as part of a broader AR serious game. This gap presents an opportunity for the development of an AR supermarket game, designed to evaluate its accessibility and usability for cognitive training for the elderly. Unlike VR, which tends to require specially designated areas and dedicated equipment, AR-based cognitive training software can be readily incorporated into current care settings, including assisted living centers, rehabilitation facilities, or even home environments. Therefore, with the lower hardware requirements of AR games, it would be more accessible while remaining engaging. Furthermore, AR applications allow elderly users to remain physically present in their familiar environment, thus preventing potential disorientation or discomfort associated with fully immersive VR.

## 3. Materials and Methods

### 3.1. Virtual Reality vs. Augmented Reality Cognitive Training

Serious games that use immersive VR have shown promise in cognitive training by providing engaging and immersive experiences. However, the required HMDs are often associated with cybersickness, which causes symptoms such as eye strain, headaches, disorientation, nausea, dizziness, and visual discomfort [17,18]. Some studies suggest that younger adults are more susceptible to cybersickness than older adults [18,19,20]. Even so, factors such as session duration, content type, hardware limitations, and underlying health conditions can increase the risk of cybersickness [18,19,20,21,22].

AR is an immersive technology that enhances reality by superimposing virtual objects onto the real-world in real time, improving immersion and interaction, resulting in potential to be applied in many areas [23]. This makes it suitable for serious games, which require minimal complexity in controls, allowing seniors to engage in interactive, accessible experiences that promote cognitive training.

AR systems can also cause cybersickness, but the severity is different compared to VR [24]. A study that compared directly AR and VR using mixed reality headsets showed that the players experienced higher levels of cybersickness in VR, which reinforces that AR platforms are more suitable for longer and more comfortable sessions [25]. Between AR systems, AR headsets can cause oculomotor disturbances with symptoms like visual discomfort, difficulty focusing, and headaches, especially in longer sessions, while AR–tablet setups are associated with lower levels of cybersickness [24].

Furthermore, HMD setups are usually more expensive and require more physical space which can reduce their practicality. For these reasons, MarketMind AR was designed specifically for Android smartphones and tablets for their lower cost, higher accessibility, and ease of use.

### 3.2. Human-Centered Design Principles

User involvement in Human-Centered Design (HCD) is a set of methods focused on creating systems with active user involvement [26]. It is pivotal for creating effective and user-friendly interfaces and can play an important role in various phases of the design process, from gathering requirements to evaluation. The user involvement can range from minimal, such as consultations and usability testing, to extensive, where users collaborate as design partners throughout the process. There are several methods that users can provide for their insights into their needs and preferences like interviews, questionnaires, prototyping, usability testing, and participatory design. Involving users in multiple stages of the development process ensures that the final design is close to their expectations [27,28].

In the context of the development of MarketMind AR, data from a review of relevant work that applied HCD principles methodology were utilized in order to ensure that the practices implemented were aligned with the needs of older adults, such as the use of large icons and fonts, simple and consistent design, sufficient time for content assimilation, alternative input formats, and the use of simple and understandable language [16,29,30,31].

To accommodate the varying cognitive abilities of older adults, MarketMind AR was designed with adaptive difficulty levels and a real-world storytelling approach. The game offers four difficulty settings (from very easy to hard) that adjust task complexity by modifying the number of shopping list items, memorization load, and available hints. By simulating a supermarket shopping experience, MarketMind AR provides contextual cognitive training, reinforcing memory, attention, and executive function through familiar daily tasks. The scoring system rewards engagement by applying difficulty-based multipliers, while feedback mechanisms emphasize positive reinforcement, ensuring a motivating and accessible experience for users with diverse cognitive abilities.

Before the main experiment, a preliminary testing phase was conducted to refine MarketMind AR mechanics and usability for older adults. This included internal testing by the development team and preliminary testing with a small group of older adults (*n* = 4) to assess interaction and technical functionality. Key aspects evaluated included AR scanning, object detection, interface usability, and interaction flow. Participant feedback on difficulty levels, shopping list memorization, PIN recall, and product selection informed refinements such as improving object selection responsiveness, enhancing interface visibility, and adjusting difficulty scaling. Additionally, the Play-In-Editor feature of Unreal Engine facilitated rapid iteration and debugging, streamlining refinements without the need for repeated APK generation.

### 3.3. MarketMind AR

**Figure 1:**
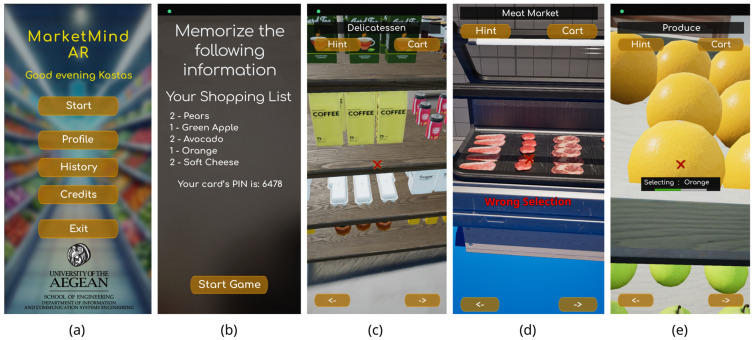
(**a**) Starting screen of MarketMind AR. (**b**) Screen with shopping list that user needs to memorize. (**c**) Shelf with delicatessen products. (**d**) Screen from meat refrigerators through trial attempt within Play-In-Editor feature. (**e**) Selecting product.

MarketMind AR (Figure 1a) is a serious AR game for the cognitive training of the elderly. It provides an interactive supermarket shopping simulation to improve attention, memory, and executive functions with the aim of training and enhancing cognitive skills. The game takes place in an AR environment, where the real space is augmented with shelves resembling those found in a supermarket. The player must use their smartphone to select items located on these shelves.

At the beginning of each game session, the player is presented with a shopping list consisting of random products found on the virtual supermarket shelves, as well as a four-digit PIN code for payment (Figure 1b). The number of products on the shopping list depends on the difficulty level selected for the specific session. The player can choose a specific difficulty level from four pre-existing options: very easy, easy, medium, and hard. The chosen difficulty level affects the number and individual target quantities of products in the list that the player must locate and acts as a multiplier in calculating the final score. The player must memorize the list of products in random quantities along with the PIN code and then find the products on the shelves. The game features various categories of shelves and refrigerators corresponding to different sections in a supermarket, such as grocery, delicatessen (Figure 1c), meat refrigerators (Figure 1d), dairy refrigerators, and others. The player can switch the shelf in front of them using arrow buttons located at the bottom of the screen. 

**Figure 2:**
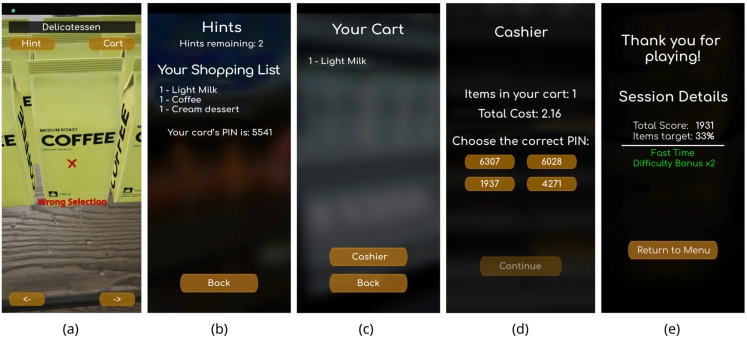
(**a**) Wrong item selection message. (**b**) Help screen. (**c**) Screen with user’s shopping cart items. (**d**) Cashier screen. (**e**) Final screen with information about player’s performance.

When the player finds a product from the shopping list, they must move close to it and aim at it with the mobile camera for a few seconds to select the item. The time required for selection is indicated by a loading bar that appears on the screen below the aiming target (Figure 1e). This approach eliminates the need for additional interfaces. If the selection is correct, the product is added to the shopping cart; otherwise, a message is displayed indicating that the selection was incorrect (Figure 2a). 

During the product selection process, the player can use the help button at the top of the screen. By selecting help, the shopping list and PIN code are displayed again (Figure 2b). Help can be used a limited number of times, which depends on the selected difficulty level. For example, at the easy difficulty level, the player has three hints available, while at the moderate difficulty level, they have four hints. The player can also check how many products they have already placed in their basket by selecting the basket option at the top right of the screen, which displays the relevant list (Figure 2c). 

When the player completes their attempt, they select the “Cashier” option from the shopping cart to proceed to the payment screen (Figure 2d). At this point, the player must recall the PIN code and select it from the four available options to complete the purchase. Upon completion, a screen appears displaying the percentage of correctly found products, the score, and additional feedback, such as whether the player completed the task quickly or entered the PIN correctly (Figure 2e). The feedback focuses on positive elements, while any negative elements are hidden and available for analysis at a later time, ensuring a positive player experience in the game.

Table 2 summarizes the key HCD specifications implemented in the MarketMind AR system to support accessibility, usability, and a seamless user experience.

### 3.4. Technological Implementation

Developers can create AR applications using Software Development Kits (SDKs) and frameworks that provide tools and functionalities for integrating AR experiences into mobile and other platforms. Two widely used SDKs are ARCore by Google and ARKit by Apple, which enable AR development for Android and iOS devices, respectively. Both are free to use and share similar core features, like motion tracking, plane detection, light estimation, and image detection, enabling the development of AR applications and the choice between them depends on the target platform for development [32,33]. For this study ARCore was selected because the target platforms are Android devices.

ARCore [34,35] supports motion tracking with simultaneous localization and mapping (SLAM). For motion tracking, ARCore uses inertial tracking by utilizing the device’s camera and Inertial Measurement Unit (IMU), which typically includes accelerometer and gyroscope sensors. This ensures that virtual objects are overlaid with the correct perspective. ARCore utilizes data from the device’s camera to generate depth maps for more accurate placement and interaction of virtual objects. If available on the device, hardware depth sensors such as Time-of-Flight cameras are also used [36]. ARCore understands the environment and detects flat surfaces, allowing virtual objects to be placed on them realistically, and with light estimation, it corrects the lighting of the virtual objects for a more natural integration into the real-world scene [34,35].

The game engine that was used for the development was Unreal Engine 5 (version 5.3). It integrates Google AR Core, which allows the development of AR environments. Additionally, the Android SDK (API level 34) was used, as it is required for developing applications on Android devices. The functions were implemented using a combination of C++ code and the Blueprints engine system and the file version control was performed with Git technology deployed on an external provider for hosting the Git server. The development was carried out on high-performance computers featuring 8-core CPUs, 32 GB DDR4 RAM, NVIDIA RTX GPU and the Windows 11 OS. The application evaluation was conducted on Android devices such as the Samsung S21 (Samsung, Suwon, Republic of Korea), Samsung S24 Plus (Samsung, Suwon, Republic of Korea), and Xiaomi Redmi Note 9 (Xiaomi, Beijing, China).

The AR template provided by Unreal Engine [37] was utilized as the starting point and it was heavily modified for the game. This template includes pre-implemented functions that showcase basic AR capabilities. The 3D models used in the game were sourced from the Unreal Engine Marketplace (now FAB [38]).

During the development of the game, a parallel verification environment was created within Unreal Engine using traditional game development functions to overcome the time-consuming process of generating and installing APK files on a test device. The AR environment was simulated in a 3D world, and Unreal Engine’s Play-In-Editor feature was utilized to quickly verify changes without requiring APK generation. Figure 1d shows a test performed within the editor in a simulated environment.

To simplify the environmental scanning process and improve the accuracy of virtual shelf placement in the deployed version, a custom Blueprint function was implemented that filters detected surfaces and automatically identifies and selects the first suitable surface. This is conducted by prioritizing horizontal and sufficiently wide areas that can accommodate the supermarket shelves and excluding small irregular surfaces.

The menu graphics were developed using Unreal Motion Graphics (UMG), Unreal Engine’s comprehensive UI implementation system. Since MarketMind AR is designed specifically for seniors, special attention was given to usability and accessibility. Large font sizes were used to enhance readability, and the menus were designed to be simple and intuitive, as outlined in Table 2.

Some of the user data are stored locally for quick access, while other data are saved in the Firebase Realtime Database for analysis. The selected difficulty level and username are stored locally in a SaveGame Object, ensuring fast retrieval. The game session data are stored in Firebase Realtime Database [39], a cloud-based NoSQL database. The collected data are used to analyze the application’s effectiveness and evaluate user performance. Each user has a unique document under the “users” collection, which contains their account creation timestamp and a “play_sessions” collection that tracks all completed game sessions. Each session document stores metrics such as accuracy percentage, difficulty level, score, time taken to complete, and bonuses for correct PIN selection and fast completion. Users can view a summary of their completed sessions by selecting “History” from the main menu, where they can access details such as score, difficulty level, and completion time for each session.

### 3.5. Evaluation Study

A study was conducted to evaluate the application with the involvement of 15 seniors. Participants were recruited based on specific inclusion and exclusion criteria to ensure suitability for the study and to represent the targeted population. The inclusion criteria required individuals to (i) be aged 60 years or older, (ii) have the ability to understand and follow study instructions, and (iii) report no significant cognitive impairment. The exclusion criteria encompassed the following: (i) the presence of neurodegenerative disorders affecting cognitive function (e.g., dementia), (ii) severe visual impairments, and (iii) motor impairments that might hinder effective game interaction. Recruitment was conducted through local senior centers, community groups, and word-of-mouth referrals. The final sample consisted of older adults with a diverse range of characteristics, as presented in Table 3.

The study followed a structured evaluation protocol, with the steps outlined as follows:

- The participants gave their consent for data collection and were informed about the purpose, relevant procedures, potential risks, benefits, and confidentiality agreements.
- A short tutorial introduced the core mechanics of MarketMind AR, ensuring that participants understood how to interact with the game using their mobile device’s sensors.
- Each participant played the game three times. The first two attempts were at the easy level, and the third attempt was at moderate difficulty, with minor adjustments if necessary to better match the players’ skill level and prevent the process from becoming tiring. A short break was given between attempts.
- For the evaluation of the game, both quantitative and qualitative methods were used, as well as some demographic data and data for familiarity with technology, in order to create the profile of the participant characteristics (Table 3). The following assessment measures were utilized after the participants had played the game three times.

The quantitative methods included three questionnaires: the System Usability Scale (SUS), the Game Experience Questionnaire (GEQ), and the Unified Theory of Acceptance and Use of Technology (UTAUT). These three questionnaires were selected for the usability and acceptability evaluation of the MarketMind AR because they are widely adopted and validated tools.

The qualitative evaluation was conducted through a semi-structured interview discussion. Performance data collected during the use of the game and stored in the database (e.g., completion time, PIN recall accuracy) were also evaluated.

#### 3.5.1. System Usability Scale (SUS)

SUS [40] is a simple tool developed by John Brooke to assess users’ subjective impressions of a system’s usability. It consists of a ten-item questionnaire with a mix of positive and negative statements rated on a 5-point Likert scale, ensuring that respondents read each item. The final SUS score ranges from 0 to 100. SUS is the most widely used measure of perceived usability and its robustness to changes and translations and SUS means corresponds with the means of other usability questionnaires [41]. The SUS questionnaire used is shown in Table 4.

SUS is a widely adopted tool for assessing perceived usability across various technological systems, including mobile applications and multimedia systems, with prior research showing that age, level of education, and type of participants do not significantly influence SUS scores [42].

#### 3.5.2. Game Experience Questionnaire (GEQ)

GEQ [43] is a comprehensive instrument designed to evaluate players’ subjective experiences during and after gameplay. It consists of three modules: the Core Module, for assessing seven key components of in-game experience, like competence, flow, and immersion; the Social Presence Module, which examines psychological and behavioral involvement with other players or virtual characters; and the Post-game Module, which evaluates players’ emotional states after gameplay. A more concise version of GEQ, the in-game version (iGEQ), enables assessment during gameplay and assesses the same key components as the Core Module and uses only two items per component. GEQ uses a 5-point Likert scale for responses ranging from “not at all” = 0 to “extremely” = 4. GEQ is widely used for evaluating player experiences, and it is broadly adopted, highlighting its effectiveness [44,45].

GEQ is widely used in player experience research across various game genres and user groups [45], and it was selected to assess player experience during gameplay, focusing on factors such as competence, immersion, flow, challenge, and affect. Unlike productivity applications, where effectiveness and efficiency are more important, games need to evaluate the gaming experience, making concepts like flow and immersion more relevant [46].

For the evaluation of this serious game, the iGEQ version was used as it is quicker and reduces the burden on the participants. The users answered after completing their third try of the game.

#### 3.5.3. Unified Theory of Acceptance and Use of Technology (UTAUT)

UTAUT identifies performance expectancy (PE), effort expectancy (EE), social influence (SI), and facilitating conditions (FCs) as the four key constructs of user behavioral intention (BI) with moderators like gender, age, experience, and voluntariness influencing these relationships [47].

Using UTAUT as a framework for understanding the adoption of a serious game by its target group, the key factors influencing user acceptance and engagement were analyzed [16]. More specifically, PE reflects the belief that playing a serious game will lead to meaningful outcomes, such as skill development, knowledge acquisition, or improved performance in real-world tasks. EE represents the perception that the serious game is easy to use and does not require significant technical effort or prior expertise. SI reflects the role of external influences, such as encouragement from peers like family or friends, in motivating individuals to engage in a serious game. FC represents the availability of resources, such as the necessary equipment, support, and guidance, that enable effective adoption and use of the serious game. Finally, BI is influenced by the four above key constructs and has a direct effect on the actual behavior of the user toward the serious game.

For this study, the participants were asked to answer questions related to PE, EE, FC, and BI using a 5-point Likert scale in an adapted questionnaire (Table 5). While social influence (SI) is a key construct in the UTAUT model, it was excluded from the questionnaire due to its limited relevance in the context of this study.

The UTAUT questionnaire was selected to assess the acceptance of MarketMind AR. The UTAUT model identifies key constructs that influence technology adoption [47]. UTAUT has been widely adopted in a wide variety of fields [48].

## 4. Results

To assess the internal consistency, Cronbach’s alpha (a) and McDonald’s Omega (ω) were calculated to assess the internal consistency of the relevant questionnaires. McDonald’s Omega (ω) is a better way to measure reliability than Cronbach’s alpha because it gives more accurate results when items vary in how much they contribute [49]. The analysis was conducted using the Psych package [50] in programming language R (version 4.4.2).

### 4.1. SUS Questionnaire

SUS demonstrated good internal consistency, with an Omega Total of 0.83 and Cronbach’s alpha of 0.81, indicating that the scale reliably measures overall usability. Most items showed strong contributions to the usability construct, with factor loadings (from the Schmid–Leiman transformation) ranging from 0.27 (S10) to 0.83 (S3). Notably, S1, S3, S6, S8, and S9 contributed strongly to the scale (λ > 0.7), which is considered strong, while S4, S5, S7, and S10 displayed weaker contributions with λ < 0.50 [51].

The median SUS score for all users is 77.5. A 95% bootstrap confidence interval (CI) calculated using 1 million resamples ranges from 67.5 to 85, which implies relatively consistent perceptions of usability among the 15 participants. Given the small sample size, the bootstrap method was used to avoid assumptions about normality.

Positive statements (odd-numbered items), which assess aspects such as ease of use and satisfaction, received higher scores ranging from 3.67 to 4.07, with a median of 4. In contrast, negative statements (even-numbered items), which assess aspects like complexity and dissatisfaction, received lower scores ranging from 1.5 to 1.8, with a median of 1.8.

The standard deviation of 10.01 for the overall SUS score indicates there were no extreme differences in user responses, while the standard deviation for each individual SUS statement (ranging from 0.6 to 0.9) shows that the answers were consistent.

**Figure 3:**
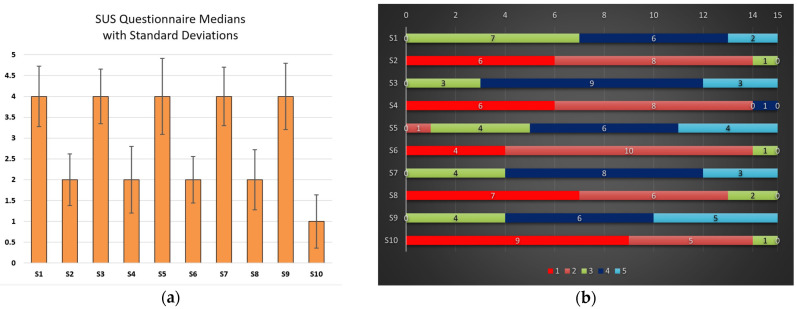
(**a**) The median score for each SUS statement with the standard deviations as error bars; (**b**) the distribution of the answers for each question.

The median SUS scores for each question and the standard deviations are presented in Figure 3a, while the graphs in Figure 3b illustrate the distribution of the responses. As shown, the positive (odd-numbered) questions typically received ratings of 3, 4, or 5, while the negative (even-numbered) questions were rated between 1 and 2.

### 4.2. GEQ

**Figure 4:**
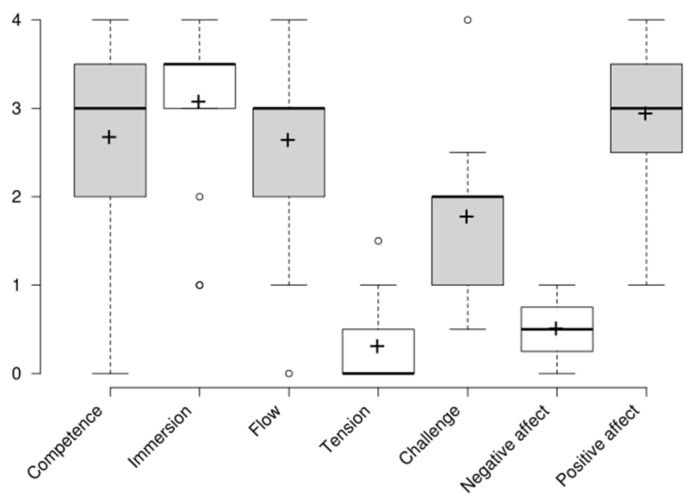
Boxplot of responses for each component of GEQ (mean scores are marked with a cross).

The reliability analysis of iGEQ revealed strong internal consistency across several metrics. Cronbach’s alpha (α) was calculated as 0.87, indicating good internal consistency, while Omega Total (ωt), a more robust measure accounting for all factor loadings, was 0.89, further supporting the scale’s reliability. This means that 89% of the variance in the total scores is due to the construct being measured, while 11% is due to random error or other factors. Most items contributed strongly to the overall construct, with factor loadings ranging from 0.43 (challenge) to 0.97 (positive affect). However, the item tension (h^2^ = 0.02) showed a very low contribution to the general construct. Overall, these results suggest that the iGEQ is a reliable tool for measuring player experiences. The results for the seven items of the GEQ are presented as boxplots in Figure 4, followed by a relevant explanation.

Participants generally reported feeling competent while playing the game, as evidenced by the relatively high median score of 3 (95% bootstrap CI: [2.0, 3.5]). This indicates that most players perceived themselves as skillful and capable during the gameplay. While there is variability in answers ranging from “not at all” (0) to “extremely” (4), the distribution suggests that most participants had a positive sense of accomplishment and success.

Immersion has a median score of 3.5 (95% bootstrap CI: [3.0, 3.5]), highlighting that most players were strongly engaged in the sensory and imaginative aspects of the game, and they were immersed in the game elements, which succeeded to capture their attention and imagination. There are some outliers (scores 1 and 2), suggesting that some participants found the game less engaging.

Flow had a median score of 3 (95% bootstrap CI: [1.0, 3.0]), which suggests that participants experienced moderate absorption in the game.

The median score of 0 suggests that the players generally did not feel tense or annoyed playing the game. The outliers of players that experienced a higher amount of tension are still low to moderate (1.5). This is a positive reflection that most players had relaxed and enjoyable gaming experiences.

The median score of 2 (95% bootstrap CI: [1.0, 2.0]) suggests a moderate score for challenge, meaning that the game presented a reasonable level of difficulty for most players. Although the range of scores and presence of outliers suggest variability in how challenge was perceived with some participants finding it easier or too difficult (4), overall, the game appears to have balance for the challenge.

Negative affect scores were low, with a median value of 0.5 (95% bootstrap CI: [0.0, 1.0]), indicating that most players did not experience frustration, boredom, or other negative emotions during the game. A few outliers, however, suggest that some participants encountered negative experiences.

Positive affect scores had a median of 3 (95% bootstrap CI: [3.0, 3.5]), suggesting that users enjoyed their experience and felt satisfaction with the game.

### 4.3. UTAUT Questionnaire

For the PE construct, the Cronbach’s alpha is 0.84 and the Omega Total is 0.91, indicating high internal consistency among the items and reliability. The standard deviation of 0.78 indicates moderate variability in responses. For the EE construct, the reliability is acceptable, with a Cronbach’s alpha of 0.75 and an Omega Total of 0.88. The standard deviation of 0.62 indicates relatively low variability in responses. The FC construct has a Cronbach’s alpha of 0.66 and an Omega Total of 0.77. While the alpha is slightly below the threshold of 0.7, combining it with the Omega indicates moderate internal consistency. The standard deviation of 0.47 reflects consistent responses. The BI construct has a Cronbach’s alpha of 0.84 and an Omega Total of 0.86, indicating good internal consistency and strong reliability. The standard deviation of 0.66 suggests moderate variability among the participants responses. Overall, the results suggest that all constructs, shown in Table 6, are well measured with minimal concerns about reliability.

**Figure 5:**
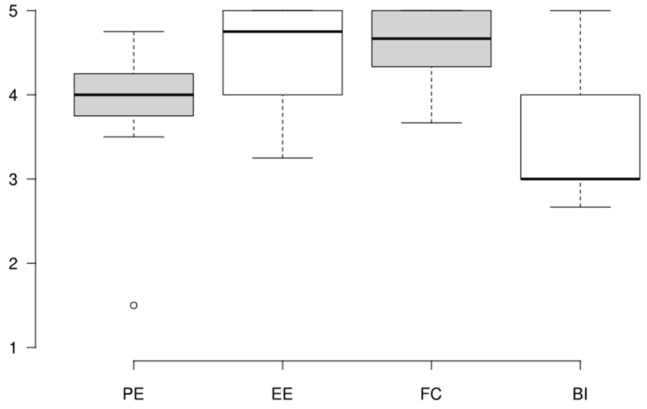
Boxplot of responses for each component of UTAUT.

The game is perceived as accessible, well supported, easy to use, and moderately beneficial, with higher scores for effort expectancy (EE) and facilitating conditions (FCs). However, the neutral score for behavioral intention (BI) suggests that engagement and excitement might not be high enough for some players, as shown in Figure 5. Due to the small sample size, the median was chosen over the mean, as it is less affected by extreme values or outliers, providing better accuracy in representing the central tendency.

More specifically, the median score of 4.00 (IQR: 3.75 to 4.25) for PE indicates that participants perceive the game as moderately beneficial. The relatively low variability further supports that participants consistently agree the game helps them achieve their goals (e.g., memory training). However, some participants (lower whisker: 3.50) might feel that the game’s utility could be improved.

For EE, the median score of 4.75 (IQR: 4.00 to 5.00) suggests that participants overwhelmingly find the game easy to use. However, lower responses (lower whisker: 3.25) indicate that some participants might experience difficulties with its usability.

For FC, the median score of 4.67 (IQR: 4.33 to 5.00) suggests that players feel well supported in accessing and using the game. However, the lower whisker of 3.67 indicates that some participants might face challenges, such as inadequate hardware or the need for additional support to fully engage with the game.

Finally, the BI construct, with a median score of 3.00 (IQR: 3.00 to 4.00), shows mixed results. While some participants exhibit high motivation (upper whisker: 5.00), others (lower whisker: 2.67) appear to lack sufficient engagement or intention to continue playing.

Spearman’s correlation analysis was also conducted in R to determine the most influential constructs because the dataset exhibited characteristics that did not satisfy the assumptions of normality, as indicated by the median values, interquartile ranges (IQRs), and whiskers from the boxplot analysis. The results of Spearman’s correlation analysis are presented in Table 7.

The analysis revealed significant relationships between BI and PE, and EE and FC. EE had the highest correlation with BI (r = 0.846, *p* < 0.01), suggesting that the ease of use influences participants’ intention to engage with the game. PE (r = 0.639, *p* < 0.05) and FC (r = 0.558, *p* < 0.05) also show positive correlations with BI, suggesting that the perceived usefulness of the game and the availability of the resources, like hardware and support, influences, to some extent, the participant’s intention to play. The correlation between PE and EE (r = 0.545, *p* < 0.05) suggests that they are moderately related with statistically significant association. Finally, the correlation between EE and FC (r = 0.443, *p* > 0.05) suggests that there is moderate positive relationship, but it is not statistically significant.

### 4.4. Qualitative Evaluation Results

To better understand the background of the participants, their familiarity with technology was first examined. One participant mentioned having no experience with smartphones or technology in general. Six participants reported having basic experience with computers for everyday tasks but noted that they do not play games. The remaining eight participants stated that they also engage in gaming, primarily enjoying puzzles, word and number games, and card games like solitaire.

Regarding first impressions, most participants shared positive feedback, describing the game as exciting, fun, and innovative. Some mentioned that it was interesting and impressive, while others highlighted the realism of the setting and its educational values. A few users initially experienced uncertainty and difficulties but later found the game useful and engaging. Overall, the participants’ experiences and acceptance of the game were positive.

Expanding on their first interaction with the game, most participants described their experience as enjoyable and exciting, particularly appreciating the immersive and interactive aspects of the AR environment. Some expressed curiosity and anxiety, especially about whether they would be able to play the game successfully. Others initially encountered difficulties but later found the experience enjoyable and engaging.

When it came to learning how to play the game, seven out of fifteen participants found it easy to understand, with some noting that the available instructions and guidance helped them adjust. However, four participants initially encountered moderate difficulty, but after receiving demonstrations and guidance, they adapted. The remaining four participants also reported moderate difficulty, particularly in understanding how to switch between shelves and select items.

While most participants were able to navigate the game with ease, about half reported no significant issues or mentioned that they easily overcame any difficulties. However, some found certain objects—particularly those on the highest and lowest shelves—difficult to reach. Additionally, two-thirds of participants encountered difficulties related to AR functionality, specifically with scanning and detecting the environment. Even when the environment was detected, some users struggled to interact with objects, and shelves sometimes shifted or moved, making it difficult to focus and collect objects. In some cases, users had to repeat the process.

When asked what aspects of the game they found most appealing, participants highlighted various elements. Most cited the realistic supermarket simulation and the immersive shopping experience within the augmented environment. Several participants enjoyed interacting with objects through augmented reality, while others praised the game’s visuals. Some also found the variety of products and the challenge of remembering them particularly engaging.

Participants provided several reasons for continuing to play the game. Many mentioned that the challenge and difficulty levels were key motivators. Some enjoyed testing their skills, tracking their progress, and successfully completing activities, while others were motivated by advancing to the next level. A few participants also appreciated the novelty and interactive elements, particularly the ability to engage with objects in the AR environment.

Regarding whether the game had an impact on cognitive abilities, three out of fifteen participants felt that it would not have any effect. However, the majority believed that the game could help improve memory and attention. Many noted that it helped them practice recalling items, such as shopping lists and PIN codes, while also encouraging mnemonic strategies. Additionally, some participants felt it provided general mental exercise.

When asked whether they had shared the game with others, most participants stated that they had not yet done so. However, a few had shared it with their spouses, and some expressed intentions to introduce it to others in the future.

While participants generally had a positive experience, some suggested improvements to enhance gameplay. The most common recommendations focused on AR functionality, particularly enhancing scanning accuracy to prevent tracking and display issues. Other suggestions included lowering the upper shelves to improve accessibility and adding a zoom feature to make interacting with objects easier. Some participants also recommended expanding the variety of products, using familiar real-world labels, and introducing more shopping scenarios. Additionally, one participant suggested hiding already-selected products in the help feature.

In terms of overall satisfaction, most participants provided positive feedback, with more than one-third reporting that they were very satisfied, highlighting the game’s fun and engaging nature. Another group described themselves as quite satisfied, though one participant noted that minor difficulties in product selection slightly affected their experience. Meanwhile, two participants gave a neutral response.

When asked whether they would recommend the game, almost all participants responded positively. Many cited cognitive benefits, such as improving memory, as a key reason for their recommendation. Others found the game enjoyable and fun, while some appreciated its technological aspect, particularly augmented reality, seeing it as a unique experience. Only one participant expressed uncertainty about recommending the game.

### 4.5. Results of Performance Metrics

**Figure 6:**
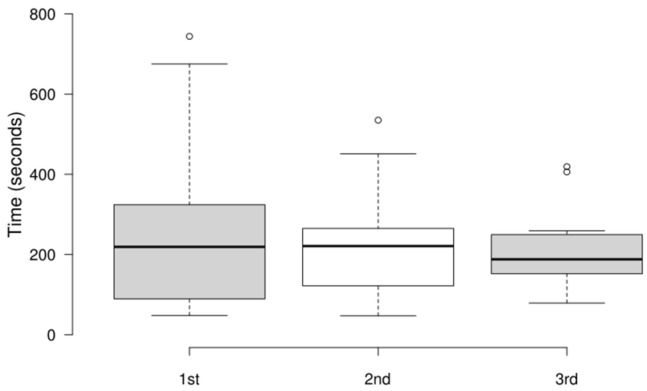
Distribution of completion times for each attempt.

This section reports the results from three gaming attempts for each of the 15 participants. Figure 6 presents the distribution of the completion time for the three attempts. The first and second attempts were easier, and the third was consistently more challenging than the other two. Despite that, the median completion time decreased from 219 s in the first attempt to 188 s in the third. The upper whisker values dropped in each of the three attempts (1st: 675 s; 2nd: 451 s; 3rd: 259 s), indicating that the slowest performers became faster over time. However, the first quartile (lower 25%) increased in the third attempt (1st: 89.5 s; 2nd: 122 s; 3rd: 152 s), as did the lower whisker (1st: 48 s; 2nd: 47 s; 3rd: 79 s), suggesting that some participants struggled in the third attempt. Overall, the results of time to completion suggest that the players adapted to the game mechanics and were able to select the items in the augmented reality more efficiently.

**Figure 7:**
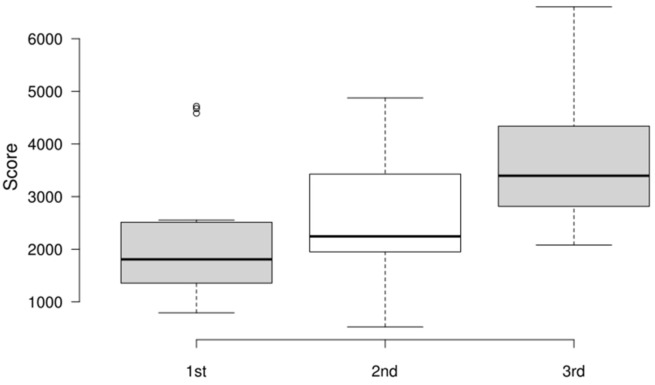
Boxplot distribution of scores across three attempts.

Analyzing the scores across the three attempts, a general improvement in performance can also be observed (Figure 7). Despite the increased difficulty in the third attempt, the median score increased from 1808 in the first attempt to 3396 in the third. The upper quartile and whisker values also show a steady rise (upper whisker: 1st: 2553; 2nd: 4874; 3rd: 6605; and 3rd quartile: 1st: 2513; 2nd: 3428; 3rd: 4339), indicating that most participants performed better in each attempt. A few participants had a drop in scores in the second attempt before recovering in the third, as shown by the values of the lower whisker (1st: 793; 2nd: 525; 3rd: 2081).

**Figure 8:**
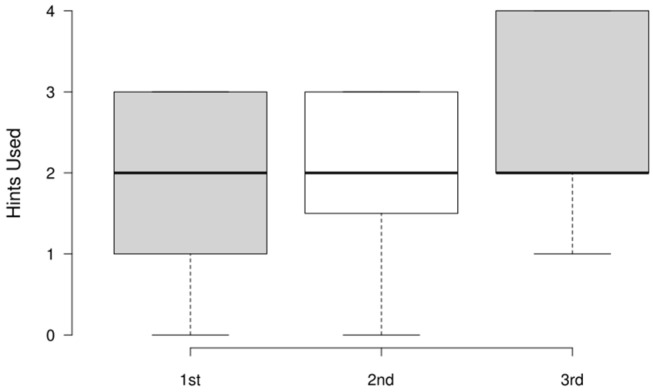
Distribution of hints used across three attempts.

The amount of hint usage across the three attempts is presented in Figure 8. In the first and second attempts, three hints were allowed, and four in the third attempt due to the increased difficulty. The number of hints used relative to the maximum allowed did not significantly increase in the third attempt. The distribution of hints used across attempts remains relatively stable and confirms that reliance did not increase proportionally.

Finally, the accuracy of PIN recall across the three attempts shows improvement, with all participants entering the correct PIN in the third attempt, while 87% and 73% did so in the first and second attempts, respectively.

## 5. Discussion

### 5.1. Usability and User Experience

The main objective of this study was to evaluate the usability, the game experience, and the user acceptance of the MarketMind AR, which is a mobile AR supermarket application designed as a cognitive training tool for older adults. The findings from the SUS, iGEQ, and UTAUT questionnaires and qualitative feedback are promising, indicating that MarketMind AR is an engaging platform for cognitive training in this target population.

SUS produced a median score of 77.5, which is indicative of “good” usability and suggests that the design choices, such as the large fonts and the simple navigation, were effective. Scores in the 70 s and 80 s indicate that the game’s acceptability is promising [7,52]. The participants’ responses to positively worded items (e.g., ease of use) and the lower scores on items assessing perceived complexity (e.g., unnecessarily complex) indicate that the design approaches were successful and appropriately tailored for the target group.

The in-game experience, as measured by the iGEQ questionnaire, further supports these usability findings. Participants reported high levels of competence, immersion, and flow, along with low levels of tension and negative affect. The overall positive affect and minimal frustration suggest that MarketMind AR captured their attention and maintained a pleasant, engaging experience.

The UTAUT results provide additional insights into the acceptance of MarketMind AR. Notably, EE demonstrated the strongest correlation with BI, indicating that ease of use is important for encouraging continued engagement. The high score in FC further suggests that participants felt well-supported through the availability of hardware, the intuitive design, and the readily available assistance. Additionally, a median score of 4 for PE combined with the average BI score indicates opportunities to further enhance the perceived benefits (e.g., the belief that the game could improve cognitive skills) to sustain long-term motivation.

The sample size of 15 participants, although relatively small, is sufficient to detect most usability problems with an 80% confidence level and approximately 80% of usability problems can be identified with a smaller group of participants [53]. This sample size is to identify usability issues and initial user feedback on engagement and technology acceptance, while future research with a larger sample and statistical power analyses will be necessary to validate the findings and assess generalizability of the results. Additionally, to assess long-term motivation and sustained engagement, future studies should extend the duration of gameplay to better capture behavioral trends over time.

### 5.2. Performance Improvements and Participants’ Feedback

The collected game session data indicate that users were able to adapt to the game mechanics and improve their performance, as reflected in faster completion times, increased scores, and enhanced PIN recall across successive attempts. Such improvements suggest possible cognitive benefits, particularly in enhancing memory and executive functions.

Qualitative feedback further supports the quantitative findings by highlighting strengths and areas for improvement. Participants found the AR supermarket to be both innovative and familiar. The familiar supermarket setting in MarketMind AR likely contributed to initial engagement by offering a recognizable and relatable environment. Familiarity can facilitate early interaction, especially among older adults and individuals with cognitive impairments; however, familiarity alone is not sufficient [54]. In this instance, familiarity may have reduced cognitive load during the initial experience by providing a context that was easy to understand based on real-world experiences, although some participants suggested the use of an even more familiar setting with the use of commonly known labels for the items. Overall, the immersive and realistic setting was appreciated. According to Bartle’s taxonomy of player types [55], most participants in this study can be characterized by their feedback as *Achievers*, as they were primarily driven by challenge, progress, and skill improvement. A smaller subset exhibited traits of *Explorers*, as they were drawn to the novelty and interactive aspects of the AR environment. However, the current design of MarketMind AR does not incorporate strong social or competitive elements, which likely explains the absence of players aligning with the *Socializer* or *Killer* categories. Addressing the challenges related to AR scanning and object detection, as noted by participants, could help minimize frustration since some experienced technical issues that may have affected their overall experience.

### 5.3. Advantages and Limitations of AR for Cognitive Training

The application of AR in cognitive training is supported by established principles of cognitive science and neuroplasticity, which highlight the brain’s ability to reorganize and strengthen neural connections through targeted mental stimulation [56]. AR enhances cognitive engagement by integrating multisensory interaction, which was shown to improve memory encoding and retrieval by activating multiple neural pathways simultaneously [57]. Unlike traditional digital cognitive training, AR fosters contextual learning, where individuals interact with virtual elements in real-world environments, reinforcing spatial awareness, executive function, and problem-solving skills [58]. Additionally, AR-based cognitive tasks promote active participation and embodied cognition, which are associated with improved learning retention and cognitive flexibility [59].

Compared to other supermarket games for older adults, MarketMind AR offers a different approach with AR instead of VR. One of the key advantages of AR is its lower hardware requirements. AR games can be played using commonly available smartphones and tablets, allowing them to be more affordable and easier to adopt, especially for older adults, compared to immersive VR cognitive training games that require expensive HMDs. The lower cost enhances the scalability, allowing for easier deployment of cognitive training games to a broader population. In addition, AR games for smartphones and tablets require a simpler setup and reduced technical complexity compared to immersive VR setups, making them better suited for use at home without requiring specialized support, thereby encouraging more frequent and consistent engagement. Beyond hardware, this approach presents comfort advantages. Applications that rely on HMDs are associated with greater risk of cybersickness [24,25], a condition that includes symptoms such as nausea, disorientation, eye strain, and dizziness [18,19,20,21,22].

The scalability as a result of the lower cost and availability of the mobile devices, the simpler setup compared to immersive VR, and the lower possibility of adverse effects like cybersickness makes MarketMind AR suitable for both healthcare and domestic use. These characteristics allow deployment across diverse settings, including homes, senior centers. or healthcare environments.

The AR-based cognitive training is less cost-effective than conventional cognitive training techniques like paper-based exercises. The same is true when compared with non-immersive computerized cognitive training. AR-based approaches, like MarketMind AR, benefit from lower hardware costs compared to immersive VR; however, they still rely on devices with sufficient processing power and camera and sensor capabilities, while non-immersive computerized cognitive training applications can run on almost any device. Furthermore, the development of AR applications involves higher complexity and development costs compared to other cognitive training tools. Despite that, AR-based training programs may complement their value with a more immersive and engaging experience that could improve adherence to the training program and justify the higher cost. Further research is required to analyze the cost-effectiveness of AR-based cognitive training compared to both conventional paper-based and non-immersive computerized approaches.

### 5.4. Challenges, Technical Limitations, and Possible Solutions

The development and evaluation of MarketMind AR demonstrated challenges related to environmental scanning, object detection, and user interaction within the augmented environment. The most important challenge observed was the inconsistency of plane detection, which led to object placement drift, where the virtual shelves occasionally shifted from their initial positions. This instability is a limitation of visual–inertial odometry (VIO) systems, such as ARCore, which estimate the device’s position by combining data from the camera and IMU and, while this approach enhances tracking accuracy under normal conditions, VIO systems remain sensitive to prolonged periods of no translational movement and to rotating the device without simultaneous forward or backward motion [60]. These limitations are relevant in MarketMind AR because the players often have pause to examine shelves or rotate their devices to search for products. Additionally, the VIO algorithm used by ARCore appears to deteriorate when the device is moved rapidly or when the game is played in low-light environments, further impacting tracking stability [61].

To mitigate these challenges, one option would be to modify how items and shelves are placed in order to reduce the need for excessive rotations of the device. Another option could be to train the participants in more effective movement techniques such as the use of slower movements and combining rotation with forward or backward movements. Ensuring that the play areas are large and well-lit could improve environmental scanning and tracking. The quality of the lighting could be evaluated with devices’ light intensity sensors to provide the user with a warning if the lighting is insufficient. Other improvements could be made by changing how the Blueprint function selects suitable surfaces. For example, the surface selection algorithm could take into account the distance of the surface from the player and the relative size between the detected surfaces. Another approach for the surface selection could be to display the detected surfaces to the user for manual selection while providing information about their quality. An option for adjusting the object detection range to allow players to select items from a greater distance could reduce the need for close physical proximity to shelves. The use of alternative AR frameworks and methods, particularly those that support marker-based tracking for anchoring virtual shelves to fixed visual references in the environment, could also be explored.

### 5.5. Future Improvements

Future work on MarketMind AR could focus on several areas. One key area for improvement is the initial plane recognition function. In addition, providing user feedback on the current lighting conditions and the quality of the detected planes could allow for corrective actions before the game starts. These enhancements could improve interaction with the augmented environment and enhance the overall user experience. Additionally, the inclusion of more gamification elements, such as personalized achievement pathways and social media sharing, could improve motivation and sustain long-term user interest. Another potential direction for future development is expanding the difficulty adjustment mechanisms. Currently, the difficulty changes are performed manually. However, machine learning models that track player behavior over time and dynamically adjust difficulty based on each player’s performance could be incorporated [62]. This could lead to a more personalized and enjoyable experience. In addition, the integration of multimodal interaction, such as voice commands and audible feedback (i.e., pronouncing aloud the name of the object the camera is pointing at), could improve accessibility, especially for older adults. Another improvement would be the development of a secure, cloud-based monitoring platform. This platform would allow caregivers and healthcare professionals to track the user’s progress and performance over time. Finally, expanding the game with additional AR-based cognitive activities could promote long-term engagement and offer a variety of exercises tailored to different cognitive functions.

Future work could adapt MarketMind AR to use with tablets and AR or mixed reality headsets to investigate whether the user experience is improved and if any limitations exist. The current version can be easily adapted for use with Android tablets. Tablets offer larger screens, which could improve visibility and thus enhance the user experience for older adults. AR and mixed reality headsets could improve immersion, engagement, and user experience, but their much higher cost makes practical use difficult. Other challenges could include comfort, as well as the relatively higher risk of cybersickness [24], especially during longer sessions.

## 6. Conclusions

This study evaluated the usability, game experience, and user acceptance of MarketMind AR. MarketMind AR is a mobile AR supermarket serious game designed for the cognitive training of older adults. For the evaluation, SUS, iGEQ, and UTAUT questionnaires, quantitative feedback, and gameplay metrics were used.

The median SUS score supports that participants found the interface user-friendly, the iGEQ findings indicate that participants found the gameplay immersive, and the UTAUT results predict the intention of participants to use this game based on the ease of use and the support. Most participants mentioned, in the qualitative evaluation, that they were satisfied and had a positive experience, although they mentioned difficulties with the placement of virtual objects. Incorporating additional gamification features could enhance the engagement. The gameplay metrics show that players can adapt to AR-based tasks.

The results indicate that usability and engagement factors significantly support cognitive training effectiveness. High usability scores, reflected in a median SUS score of 77.5 and strong EE ratings (median = 4.75), correlate with increased engagement and BI (r = 0.846, *p* < 0.01). Furthermore, participants demonstrated performance improvements in key cognitive areas, with task completion time decreasing by 14.2% (indicating increased efficiency), game scores increasing by 87.8% (reflecting better decision-making and task execution), and PIN recall accuracy improving by 37.0% (suggesting enhanced memory retention). These findings suggest that when usability facilitates intuitive interaction and engagement, cognitive training outcomes improve, reinforcing the importance of designing accessible and engaging interventions for older adults.

MarketMind AR has the potential to be used for cognitive training, but further testing with a larger group of participants is required to validate its effectiveness and long-term impact.

## Figures and Tables

**Table 1:** A summary of the studies in terms of game type, the equipment used, and the usability/acceptability evaluation methods and results for the reviewed supermarket and market-like serious games.

Study	Game Type	Main Equipment	Game Engine	Domain	Usability/Acceptability Evaluation Methods	Usability/Acceptability Results
[10]	Immersive VR	HTC Vive Pro	Unity	Cognitive Training	TAM3, ITC-SOPI, SSQ, performance metrics	high acceptability and negligible side effects
[11]	Immersive VR	HTC Vive HMD and HTC Vive Lighthouse	Unity	Cognitive Assessment	no formal scales used; based on researcher observations, performance metrics	VR system provided realistic and immersive experience
[12]	Immersive VR	Oculus Quest 2	Unity	Cognitive Assessment	no formal scales used; based on researcher observations, performance metrics	positive user experience through design choices, motion sickness reduced by seated design
[13]	Non-immersive VR	Android Tablets	Unity	Cognitive Training	custom questionnaire (5-point Likert scale)	entertaining, useful for mental stimulation, and intuitive
[14]	Non-immersive VR	Desktop PC with WebGL compatible web browser	Unity	Cognitive Assessment and Cognitive Training *	custom questionnaire (10-point Likert scale), qualitative data, performance metrics	high usability with some issues
[15]	Immersive VR	HTC Vive HMD and HTC Vive Lighthouse	Unity	Cognitive Assessment andCognitive Training *	no formal scales used; based on researcher observations, performance metrics	some interaction difficulties
[16]	AR and VR (immersive and non-immersive)	Kinect 2.0, Oculus Quest 2, and ARCore compatible Android Devices	Unity and Unreal Engine 4	Cognitive Training	SUS, VRSQ, UTAUT, qualitative data	high acceptability, enjoyable experience, good usability, low VR sickness
this work	AR	ARCore compatible Android Devices	Unreal Engine 5	Cognitive Training	SUS, in-game GEQ, UTAUT, qualitative data, performance metrics	good usability, high engagement, some difficulties with scanning, positive qualitative feedback

* Future work. HMD: Head Mounted Display; GEQ: Game Experience Questionnaire; SSQ: Simulator Sickness Questionnaire; ITC-SOPI: International Test Commission—Sense of Presence Inventory; TAM3: Technology Acceptance Model; SUS: System Usability Scale; VRSQ: Virtual Reality Sickness Questionnaire; UTAUT: Unified Theory of Acceptance and Use of Technology.

**Table 2:** Human-Centered Design specifications in MarketMind AR.

Specification	Implementation in MarketMind AR
Large, clear icons	Buttons are large, with clear textual descriptions instead of icons. White, high-contrast text ensures readability and a larger touch target.
Large font sizes, sans-serif style (e.g., Arial), no italics, underlines, or all caps	The font is large, with uniform thickness, and avoids special styles.
Simple and consistent layout	Consistent button colors (brown/mustard with white text), stable text alignment, and uniform spacing.
High contrast between background and foreground colors	Important UI elements, like the “X” targeting icon, have a bright yellow border for visibility, while the charging bar has a black background.
Avoid text within images	If text in images is necessary, a blurred dark tint is applied to ensure readability.
Multimodal information delivery	Information is conveyed through 3D graphics, text, and auditory feedback.
Adequate time for content absorption	No visible countdown timer; players can take their time without stress.
Minimize text input fields	The only text input field is for the username; other inputs use buttons and selection lists.
Avoid scrolling	No scrolling is required, except in the game history screen when necessary.
Simple and understandable language	Text is clear and formal (politeness register).
Easy navigation	Menus are designed for ease of use with minimal interactions required.
Encouraging feedback in games	Feedback is limited to completion percentages, scores, and positive reinforcement. Negative feedback is avoided except when a wrong item is selected, where an explanatory message is shown.

**Table 3:** Participants’ characteristics (*n* = 15).

Characteristic	Value
Age (Mean, Stdev)	66.5 ± 3.5
Gender (Male/Female)	9/6
Education: High school/technical school/Bachelor’s degree	6/2/7
Full time employed/retired	3/12
Residential area: Urban/semi-urban/ rural	10/3/2
Participation in social activities: Not at all/rarely/sometimes/often/very often	1/2/4/4/4
Access to digital technologies: Sometimes/often/very often	2/11/2
Experience with serious games (Yes/No)	2/13

**Table 4:** SUS questionnaire for MarketMind AR.

ID	Statement
S1	I think that I would like to use this game frequently.
S2	I found the game unnecessarily complex.
S3	I thought the game was easy to use.
S4	I think that I would need the support of a technical person to be able to use this game.
S5	I found the various functions in this game were well integrated.
S6	I thought there was too much inconsistency in this game.
S7	I would imagine that most people would learn to use this game very quickly.
S8	I found the game very cumbersome to use.
S9	I felt very confident game the system.
S10	I needed to learn a lot of things before I could get going with this game.

**Table 5:** UTAUT-based questionnaire for MarketMind AR acceptance assessment.

Construct	Item Code	Item
Performance Expectancy	PE1	I find MarketMind AR useful as a simulation of my daily life activities.
	PE2	Using MarketMind AR can potentially improve my cognitive skills.
	PE3	Using MarketMind AR helps me improve my mood.
	PE4	Using MarketMind AR increases my self-confidence.
Effort Expectancy	EE1	Learning to use MarketMind AR is easy for me.
	EE2	My interaction with MarketMind AR is clear and understandable.
	EE3	I find MarketMind AR easy to use.
	EE4	It is easy for me to become proficient in using MarketMind AR.
Facilitating Conditions	FC1	I have the necessary resources to use MarketMind AR.
	FC2	I have the necessary knowledge to use MarketMind AR.
	FC3	I can ask for help from others when I have difficulties using MarketMind AR.
Behavioral Intention	BI1	I plan to continue using MarketMind AR in the future.
	BI2	I foresee using MarketMind AR in my life.
	BI3	I plan to continue using MarketMind AR frequently.

**Table 6:** Descriptive statistics with Cronbach’s alpha and McDonald’s Omega Total scores for individual constructs in UTAUT model (*n* = 15).

Construct	Cronbach’s Alpha	McDonald’s Omega Total	Mean	Median	95% Bootstrap CI	Std. Deviation (SD)
PE	0.84	0.91	3.9	4	[3.75, 4.25]	0.778
EE	0.75	0.88	4.42	4.75	[4, 5]	0.624
FC	0.66	0.77	4.51	4.67	[4.33, 5]	0.469
BI	0.84	0.86	3.42	3	[3, 4]	0.660

**Table 7:** Correlation matrix of UTAUT constructs (*n* = 15).

	PE	EE	FC	BI
**PE**	1.000			
**EE**	0.545 *	1.000		
**FC**	0.351	0.443	1.000	
**BI**	0.639 *	0.846 **	0.558 *	1.000

The values with two bold asterisks, **, indicate that the correlation is significant at *p* < 0.01, whereas one asterisk, *, indicates that the correlation is significant at *p* < 0.05.

## Data Availability

Data are available upon reasonable request to the corresponding author.

## Abbreviations

The following abbreviations are used in this manuscript:

AR	augmented reality
BI	behavioral intention
CI	confidence interval
EE	effort expectancy
FCs	facilitating conditions
GEQ	Game Experience Questionnaire
HCD	Human-Centered Design
HMD	Head Mounted Display
iGEQ	in-game Game Experience Questionnaire
IMU	Inertial Measurement Unit
IQR	interquartile range
MCI	Mild Cognitive Impairment
PE	performance expectancy
SI	social influence
SLAM	simultaneous localization and mapping
SUS	System Usability Scale
UI	User Interface
UTAUT	Unified Theory of Acceptance and Use of Technology
VIO	visual–inertial odometry
VR	virtual reality
